# The Beit Fellowships for Medical Research

**Published:** 1910-03-05

**Authors:** 


### The Beit Fellowships for Medical Research.

At the meeting of trustees of the Beit Memorial Fellowships, held on February 23, the first election of Beit Fellows was made. Out of no less than 70 applications received, ten were selected for final consideration, and these were successful in obtaining fellowships. In glancing over the list of newly elected research students it is at once evident that the trustees exact a high standard of excellence in the applicants, but it is also evident that they pay great attention—one might almost say too great attention—to theoretical subjects for research. It is true there are some excellent practical subjects proposed and sanctioned for investigation. Dr. Otto May will devote himself exclusively to the clinical and experimental research on peripheral nerve lesions; Dr. Lewis to the mechanism of cardiac irregularities; Mr. Hindle to the morphology and treatment of protozoic blood parasites; and Dr. Edridge-Green to problems of colour vision. All these are excellent subjects for investigation. The other Fellows have not been so fortunate. Dr. Mellanby proposes to investigate " the significance of the large excretion of creatin in cancer of the liver and its diminished excretion in cirrhosis of the liver; Dr. Ransom " the action of caffeine, theobromine, and allied substances on the muscular and nervous systems "; Mr. Sydney Russ " the association of radio-activity with cancer "; Miss Smedley (the only woman who has gained a fellowship) the processes involved in the formation of fat in the organism; Dr. Mathison certain physiological questions, and Mr. Drew the zoological distribution of cancer. It will be seen that of the Fellows those who propose devoting themselves to subjects of practical interest are comparatively well known, while the others, whose ambitions remind one very strongly of those that obsess the Academicians of Lagado, are as comparatively unknown. There must of necessity be some difference of opinion regarding the value of these various subjects selected for investigation, and we should be very much surprised if anyone is daring enough to suggest that they are all of equal or relatively equal importance. We can imagine no subjects more thoroughly worthy of attention than that which Dr. Edridge-Green and Dr. May propose to investigate, and we shall look forward to their reports with great interest. On the other hand, we fail to see the urgent need for further investigation into fat metabolism such as Miss Smedley undertakes, and we are entirely sceptical of the value of such work as Dr. Mellanby proposes. The popularity of cancer subjects is a sign of the times; we can only regret that none of the investigators seem to be drawn to the clinical side of the case. It is interesting to note that two of the new Fellows are medical men in active practice. By the terms of the trust deed the Fellows are required to devote their whole time to the research they have undertaken, and there can be no doubt that this proviso prevented some medical men at least from sending in their applications. It would be desirable for the trustees to clear up this point, and state definitely whether or not a Beit Fellow is able to hold his fellowship jointly with hospital or other appointments.

